# Influence of probiotic supplementation on the growth performance, plasma variables, and ruminal bacterial community of growth-retarded lamb

**DOI:** 10.3389/fmicb.2023.1216534

**Published:** 2023-07-27

**Authors:** Huiling Mao, Wenwen Ji, Yan Yun, Yanfang Zhang, Zhefeng Li, Chong Wang

**Affiliations:** ^1^College of Animal Science and Technology, College of Veterinary Medicine, Zhejiang A & F University, Lin'an, China; ^2^Key Laboratory of Applied Technology on Green-Eco-Healthy Animal Husbandry of Zhejiang Province, Hangzhou, China; ^3^Hangzhou Kingtechina Feed Co., Ltd, Hangzhou, China

**Keywords:** growth-retarded lamb, growth performance, probiotic, plasma variables, ruminal bacterial community

## Abstract

**Introduction:**

Growth-retarded lambs would reduce the economic incomes of sheep farming. Nutritional interventions are supposed to promote gastrointestinal health and the compensatory growth of growth-retarded lambs. This study evaluated the effects of probiotic supplementation on the growth performance, plasma characteristics and ruminal bacterial community of growth-retarded lambs.

**Methods:**

Twenty-four 50-days old male Hu lambs, including 8 healthy lambs (13.2 ± 1.17 kg) and 16 growth-retarded lambs (9.46 ± 0.81 kg), were used in this study. The 8 healthy lambs were fed the basal diet and considered the positive control (GN), and the other 16 growth-retarded lambs were randomly assigned into 2 groups (basal diet without probiotic [negative control, GR] and basal diet supplementation with 1 g/kg concentrate feed probiotic [GRP]), with each group having 4 replicate pens. The feeding trial lasted for 60 days with 7 days for adaptation.

**Results:**

The results showed that dietary supplementation with probiotic increased (*p* < 0.05) the average daily gain and dry matter intake of growth-retarded lambs. For growth-retarded lambs, supplementation with probiotic increased (*p* < 0.05) the activities of superoxide dismutase and glutathione peroxidase, as well as the concentrations of growth hormone and immunoglobulin G. Furthermore, the highest (*p* < 0.05) concentrations of interleukin-6, interferon-gamma and tumor necrosis factor alpha were observed in the GR group. The concentrations of total volatile fatty acids and acetate in growth-retarded lambs were increased by probiotic supplementation (*p* < 0.05). The relative abundances of *Ruminococcus, Succiniclasticum* and *Acidaminococcus* were lower (*p* < 0.05) in growth-retarded lambs. However, probiotic supplementation increased (*p* < 0.05) the relative abundances of these three genera.

**Discussion:**

These results indicate that dietary supplementation with probiotic are promising strategies for improving the growth performance of growth-retarded lambs by enhancing immunity and altering the ruminal microbiota.

## 1. Introduction

In the current intensive production system, a certain number of lambs are growth-hindered for various reasons, resulting in “growth-retarded lambs.” Severe malnutrition during the gestation or neonatal periods (Du et al., 2010), illness followed by pneumonia or coughing, and infection with parasites can restrict the healthy development and growth of animals (Gu et al., 2017; Ma et al., 2020b). The growth-retarded lambs with low body weight and feed conversion rate, high morbidity and mortality could reduce the economic incomes of sheep farming, thus, promoting the compensatory growth of growth-retarded lambs is an urgent issue to be addressed.

The healthy development of the gastrointestinal tract plays an important role in nutrient absorption, immune system development and healthy growth of animals (Celi et al., 2017). Zhang et al. (2018) demonstrated that the intestinal development was delayed and intestinal integrity, immune function, and oxidative status were impaired in intrauterine-growth-retarded suckling lambs. A study on the growth-retarded yaks revealed that they had lower rumen weight compare to the normal yaks (Hu et al., 2016). Ma et al. (2020a) inferred that the gastrointestinal development of growth-retarded yaks was dysplastic, as evidenced by the lower papillae height and surface area of the rumen. Severe malnutrition during the early lives of animals do harm for the structure, functions and microflora of gastrointestinal tract, and subsequently restricts the growth of animals (Meyer and Caton, 2016; Zhang et al., 2018). Looking for an effective additive to promote the gastrointestinal tract development may be a feasible method to improve the compensatory growth of growth-retarded lambs. On the other hand, the intestinal microbiota has a critical effect on regulating gastrointestinal homeostasis and function of host. Parker et al. (2017) reported that the stability of the microbial community is beneficial for the host. Ma et al. (2021a) found that modulating the microbiota (increase the relative abundances of *Ruminococcaceae, Treponema, Clostridiaceae, Prevotellaceae*, and *Lachnospiraceae*, which were related to oligosaccharide, starch, and cellulose degradation) in gastrointestinal of growth-retarded yaks could effectively enhance the digestibility, gastrointestinal fermentation and digestive enzyme activity. Considering the complex interplay between gut microbiota and host health, interventions based on the modulation of gut microbiota might be one of the potential strategies (Xie et al., 2022).

Currently, probiotics are supposed to have beneficial effect on the host health (World Health Organization, 2001) when they are supplemented with proper amounts. They are used as a safe additive to rebalance the intestinal microflora, prevent diseases, improve inflammation and digestion, and promote growth of animals (Riggs et al., 2006; Kalil, 2014). *Lactobacillus* spp., *Saccharomyces* spp., *Enterococcus* spp., and *Bacillus* spp. are widely used probiotic supplements in livestock and poultry production (Markowiak and Śliżewska, 2018). Guo et al. (2020) revealed that *Lactobacillus* sp. is one of the most important bacterial direct-fed microbial used as a growth promoter for ruminants. They have been identified to compete for nutrients in the gastrointestinal and produce antibacterial compounds, which allowed them to occupy specific niches of the gastrointestinal mucosa and activate the innate immune system of young ruminants (Frizzo et al., 2011). Moreover, *Bacillus subtilis* species were found to improve intestinal health of livestock by regulating immune function to protect against pathogenic challenge (Rolfe, 2000). A study conducted on the effect of *B. subtilis* on Duhan lambs demonstrated that *B. subtilis* altered the rumen fermentation pattern and increased the growth performance of lambs (Gao et al., 2023). Du et al. (2018) found that supplementation with probiotics (especially *Bacillus* spp.) can stabilize the intestinal microbiota of growth-retarded calves and improve their growth performance. In a review paper of Chapman et al. (2011), it was suggested that multi-strain probiotics appear to show greater efficacy than single strains. And Røssland et al. (2005) found that coculture of *Lactobacillus* with *Bacillus cereus* could stimulate the biosynthetic capacities of lactic acid strains.

Consequently, we hypothesized that nutritional intervention used to regulate the gastrointestinal microbiota, especially the rumen microbiota, would promote gastrointestinal health and the compensatory growth of growth-retarded lambs. In this study, a probiotic (co-fermented by *Lactobacillus acidophilus* and *Bacillus subtilis*) product was supplemented to the basal diet to detect their effects on the growth performance, plasma indices and ruminal bacterial community of growth-retarded lambs. The main objective of the current study is to provide insights into the effective nutritional intervention and its potential mechanisms for promoting the compensatory growth of growth-retarded lambs.

## 2. Materials and methods

### 2.1. Probiotic preparation

A probiotic product, provided by Zhejiang KangwanDechuan Technology Co., Ltd. (Shaoxing, China), is co-fermented by *Lactobacillus acidophilus* (≥1.0 × 10^9^ CFU/g) and *Bacillus subtilis* (≥1.0 × 10^9^ CFU/g). It was in powder form, so that the probiotic was mixed with the concentrate and fed to lambs.

### 2.2. Animals, diets, and experimental design

**Figure 1:**
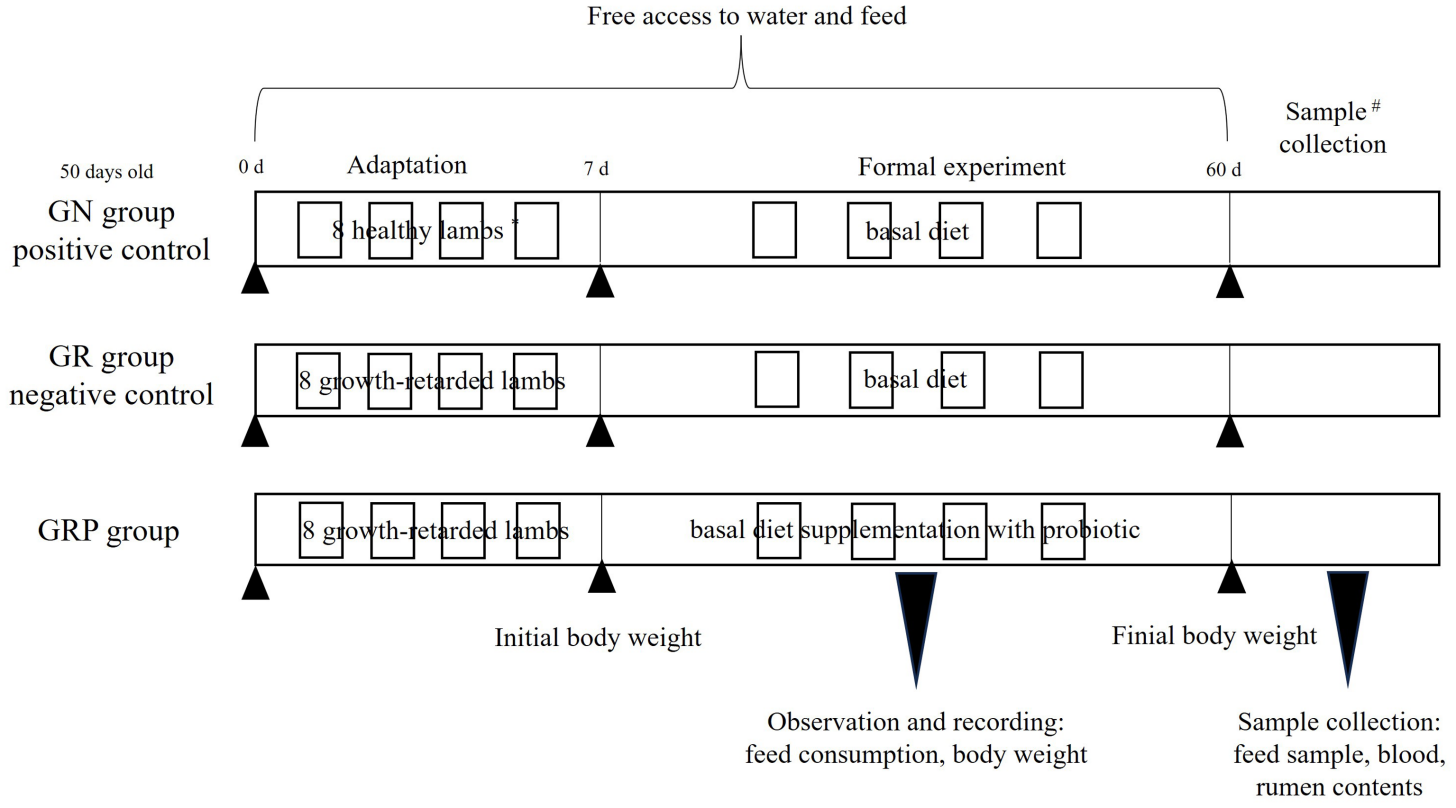
Animal and experimental design. *Two lambs with similar BWs within each group were placed and housed in one pen, which was considered an experimental replicate. Thus, there were four replicates in each treatment (*n* = 4). #One lamb of each replicate in the three groups was randomly selected to collect the blood and rumen samples. GN, growth normal lambs; GR, growth-retarded lambs; GRP, growth-retarded lambs supplemented with probiotic.

In this study, lambs with body weight at least 10th percentile below the same age lambs were defined as growth-retarded lambs. Twenty-four 50-days old male Hu lambs, including 8 healthy lambs (13.2 ± 1.17 kg) and 16 growth-retarded lambs (9.46 ± 0.81 kg), were used in this study. The 8 healthy lambs were fed the basal diet and considered the positive control (GN), and the other 16 growth-retarded lambs were randomly allotted to GR (the negative control, basal diet without probiotic) or GRP groups (basal diet supplementation with probiotic). The dosage of probiotic was 1 g/kg concentrated feed, which was recommended by the manufacturer. Briefly, 2 lambs with similar BWs within each group were placed and housed in one pen (0.6 × 1.2 m), which was considered an experimental replicate. Thus, there were four replicates in each treatment (*n* = 4). The feeding trial lasted for 60 days with 7 days for adaptation. The basal diet contained 40% concentrated feed and 60% mixed forages. The concentrated feed was purchased from AB Agri China (Anhui, China) and consisted of corn, soybean meal, wheat bran, soybean hull, stone powder, NaCl, CuSO_4_, MnSO_4_, ZnSO_4_, VA, VD_3_ and VE. The mixed forage consisted of (per 100 g DM) 14 g wheat straw, 9 g peanut straw, 35 g silage corn, and 42 g soybean curb residue. The concentrate feed and mixed forages were fed individually and their chemical compositions are shown in Table 1. All lambs had free access to water and feed. The amount of feed offered and refused were recorded every week. All individual lambs were weighted every week before the morning feeding. The schematic diagram for the animals and experimental design was present in Figure 1.

**Table 1:** Chemical composition of the basal diet (g/kg, dry matter basis)^1^.

Items	Concentrated feed^2^	Mixed forages^3^
Dry matter	891	390
Crude protein	183	69.1
Neutral detergent fiber	411	566
Acid detergent fiber	128	377
Ash	84.2	130
Calcium	11.9	8.0
Phosphorus	6.6	2.4

^1^The chemical compositions were all measured values. ^2^The concentrated feed was purchased from AB Agri China (Anhui, China) and consisted of corn, soybean meal, wheat bran, soybean hull, stone powder, NaCl, CuSO_4_, MnSO_4_, ZnSO_4_, VA, VD_3_, and VE. ^3^The mixed forage consisted of (per 100 g DM) 14 g wheat straw, 9 g peanut straw, 35 g silage corn, and 42 g soybean curb residue.

### 2.3. Feed analysis

The feed samples were dried in a forced-air oven at 65°C for 48 h, and analyzed the contents of DM (method 924.05; Association of Official Analytical Chemists, 1990), CP (method 988.05; Association of Official Analytical Chemists, 1990), acid detergent fiber (ADF expressed inclusive of residual ash, method 973.18; Association of Official Analytical Chemists, 1990), ash (method 942.05; Association of Official Analytical Chemists, 1990), calcium (method 927.02; Association of Official Analytical Chemists, 1990), phosphorus (Combs and Satter, 1992), neutral detergent fiber (NDF assayed without a heat stable amylase and expressed inclusive of residual ash) was determined according to procedure described by Mertens (2002).

### 2.4. Blood analysis

At the end of the feeding trial, one lamb of each replicate in the three groups was randomly selected to collect the blood samples (*n* = 4) before morning feeding. Approximately 10 ml blood samples were collected from the jugular vein into individual heparinized tubes, then plasma were separated by centrifuging at 3,000 × *g* at 4°C for 15 min and stored at −20°C until analysis.

The plasma concentrations of total antioxidant capacity (T-AOC, A015-2-1), superoxide dismutase (SOD, A001-3-2), glutathione peroxidase (GSH-Px, A005-1-2), and malondialdehyde (MDA, A003-4-1) were determined using commercial kits (Jiancheng, Nanjing, China). The concentrations of immunoglobulin G (IgG, JN21687), tumor necrosis factor alpha (TNF-α, JN20906), interferon gamma (INF-γ, JN20780), interleukin-6 (IL-6, JN21752) and growth hormone (GH, JN13713) were detected using ELISA kits (Jining, Shanghai, China).

### 2.5. Rumen sample collection and fermentation characteristics determination

At the end of the feeding trial, rumen fluid samples (approximately 30 ml from those lambs used to collect blood samples) were collected through rumen intubation before the morning feeding (*n* = 4), with the first 15 ml of the rumen fluid samples were discarded to avoid saliva contamination (Morsy et al., 2021). Then the rumen fluid samples were strained through four layers of compress gauze. Approximately 20 ml samples were stored at –20°C to determine the rumen fermentation characteristics; and the other 10 ml samples were stored at –80°C to analysis the ruminal bacterial community.

Ammonia-N (NH_3_-N) concentration was determined using steam distillation into boric acid and titration with dilute hydrochloric acid. Volatile fatty acids (VFA) concentration was determined by gas chromatography (Agilent 7890B, NYSE: A, Palo Alto, America) according to the method described by Hu et al. (2005). Concentrations of the microbial protein (MCP) were estimated using the purine method (Makkar and Becker, 1999).

### 2.6. Ruminal microbiota analysis

Total DNA of the rumen fluid samples were extracted by using the E.Z.N.A.® Soil DNA Kit (Omega Bio-Tek, Norcross, GA, USA). The hypervariable V3-V4 region of the 16S rRNA gene was amplified using universal primers 338F (5′-ACTCCTACGGGAGGCAGCA-3′) and 806R (5′-GGACTACHVGGGTWTCTAAT-3′). PCR products were quantified with the Quant-iTPicoGreen dsDNA Assay Kit (Invitrogen, Carlsbad, USA). The amplicon libraries were generated by pooling in an equal ratio and sequenced using paired-end protocol (2 × 300) on an Illumina MiSeq platform at Personal Biotechnology Co. Ltd. (Shanghai, China).

Microbiota bioinformatics were performed with QIIME 2 (Bolyen et al., 2018). Denoise-paired method with DADA2 was performed to filter out the quality-filtered, denoised, merged, and chimeric sequences. Amplicon sequence variants (ASVs) were clustered at 97% similarity using the Greengenes 16S reference database (gg_13). Alpha diversity measurements included Chao1 (Chao, 1984), Shannon (Shannon, 1948), Faith’s PD (Faith, 1992) and Pielou’s evenness (Pielou, 1966) were calculated by QIIME 2. Shared and unique species among groups were used to generate a Venn diagram. Principal coordinate analysis (PCoA) based on weighted UniFrac distances (Lozupone et al., 2007) was performed to compare the dissimilarity of microbiota shaped by probiotic supplementation. Analysis of similarities (ANOSIM) was used to detect the significance of microbial community differences among groups. Significant difference was declared at *R* > 0.5 with *p* < 0.05, whereas 0.3 < *R* < 0.5 with *p* < 0.05 was considered as a trend, and no difference was declared at *R* < 0.3. The linear discriminant analysis effect size (LEfSe) method (Segata et al., 2011) was used to identify bacterial taxa with significant differences among three groups. Taxa with linear discriminant analysis score greater than 3 was identified as an important contributor to each group. Besides, co-occurrence was generated based on the Spearman correlation coefficients between the major ruminal microbiota (top 20 genera) and phenotypes (rumen fermentation characteristics and plasma indices). Visualization of the co-occurrence network was performed by the genes cloud tools, a free online platform for data analysis.^1^

### 2.7. Statistical analysis

The results were statistically analyzed as a completely randomized design. Each pen with 2 lambs was considered as one experimental unit for the analysis of BW and DMI, while each randomly selected lamb from each pen was regarded as one experimental unit for the analysis of plasma variables, rumen fermentation characteristics and ruminal bacterial community. Variables of growth performance, plasma variables and rumen fermentation parameters in GN, GR and GRP groups were analyzed with one-way ANOVA procedure of the SAS statistical software (version 9.0, SAS Inst. Inc., Cary, NC, United States). Duncan’s comparison was used to determine the differences among the groups. The microbial data including alpha-diversity index and bacterial relative abundance (phylum and genus level) was compared using Kruskal–Wallis test. Statistical significance was present at *p* ≤ 0.05, and 0.05 < *p* ≤ 0.10 was declared as a tendency.

## 3. Results

### 3.1. Growth performance

During the feeding trial, 3 growth-retarded lambs from the GR group and 1 growth-retarded lamb from the GRP group died, the causes of death were present in Table 2. Compared with the GN group, the initial BW of the GR and GRP groups were significantly lower (*p* < 0.05, Table 3). However, supplementation with probiotic increased the BW of the GRP group, and there was no difference between the GN and GRP groups’ final BW (*p* > 0.05). Dietary probiotic supplementation increased (*p* < 0.05) the average daily gain (ADG) of the GRP group compared with the GR group. The lambs in the GR group had the lowest (*p* < 0.05) dry matter intake (DMI).

**Table 2:** Death causes of growth-retarded lambs.

Group^1^	Replicate	Cause of death
GR	1	Cough, pneumonia
GR	3	Diarrhoea
GR	4	Diarrhoea
GRP	1	Diarrhoea

^1^GR, growth-retarded lambs; GRP, growth-retarded lambs supplemented with probiotic.

**Table 3:** Effect of probiotic supplementation on the growth performance of growth-retarded lambs.

Items	Treatments^1^			SEM	*p*-value
	GN	GR	GRP		
Initial body weight, kg	13.2^a^	9.77^b^	9.22^b^	0.468	0.002
Final body weight, kg	27.9^a^	19.8^b^	25.2^a^	0.845	0.002
Average daily gain, g/d	245^a^	168^b^	266^a^	20.5	0.042
Dry matter intake, g/d	958^a^	534^c^	769^b^	24.1	0.0001
Feed efficiency, ADG/DMI	0.26	0.32	0.35	0.039	0.356

^1^GN, growth normal lambs; GR, growth-retarded lambs; GRP, growth-retarded lambs supplemented with probiotic. ^a,b,c^Mean values in columns without a common letter are significantly different (*p* < 0.05).

### 3.2. Plasma characteristics

For growth-retarded lambs, supplementation with probiotic increased (*p* < 0.05; Table 4) the activities of SOD and GSH-Px, while there was no difference between the GN and GRP groups (*p* > 0.05). The MDA concentration was higher (*p* < 0.05) in growth-retarded lambs; however, probiotic supplementation decreased (*p* < 0.05) the MDA concentration. Compared with the GN and GRP groups, lower (*p* < 0.05) concentrations of GH and IgG and higher (*p* < 0.05) concentrations of IL-6, IFN-γ and TNF-α were observed in the GR group.

**Table 4:** Effects of probiotic supplementation on the plasma concentrations of oxidative stress indicators and immunity variables in growth-retarded lambs.

Items^2^	Treatments^1^			SEM	*p*-value
	GN	GR	GRP		
Antioxidant					
T-AOC, mmol/L	0.13	0.13	0.13	0.006	0.869
SOD, U/ml	121^ab^	115^b^	132^a^	3.6	0.024
GSH-Px, U/ml	124^a^	91^b^	120^a^	8.5	0.044
MDA, mmol/L	3.47^c^	5.90^a^	4.90^b^	0.277	0.0006
Immune characteristics					
GH, μg/L	34.3^b^	32.2^c^	38.7^a^	0.43	<0.0001
IgG, μg/ml	1362^b^	1271^c^	1484^a^	18.9	<0.0001
IL-6, ng/L	156^b^	181^a^	161^b^	3.2	0.0009
IFN-γ, ng/L	44.7^b^	48.8^a^	46.0^b^	0.65	0.0052
TNF-α, ng/L	1287^b^	1584^a^	1349^b^	20.5	<0.0001

^1^GN, growth normal lambs; GR, growth-retarded lambs; GRP, growth-retarded lambs supplemented with probiotic. ^2^T-AOC, total antioxidant capacity; SOD, superoxide dismutase; GSH-Px, glutathione peroxidase; MDA, malondialdehyde; GH, growth hormone; IgG, immunoglobulin G; IL-6, interleukin-6; IFN-γ, interferon-gamma; TNF-α, tumor necrosis factor alpha. ^a,b,c^Mean values in columns without a common letter are significantly different (*p* < 0.05).

### 3.3. Rumen fermentation profile

For growth-retarded lambs, supplementation with probiotic increased the concentration of total VFA and acetate (*p* < 0.05; Table 5), while there was no difference between the GN and GRP groups (*p* > 0.05). The concentration of butyrate was greater in the GRP group than those of GR group (*p* < 0.05). For growth-retarded lambs, supplementation with probiotic decreased the concentration of NH_3_-N (*p* < 0.05). The concentration of MCP was the lowest in the GR group, while there was no difference between the GN and GRP groups (*p* > 0.05).

**Table 5:** Effect of probiotic supplementation on the rumen fermentation characteristics of growth-retarded lambs.

Items	Treatments^1^			SEM	*p*-value
	GN	GR	GRP		
VFA concentration, mmol/L					
Total	47.9^a^	29.9^b^	52.5^a^	2.668	0.001
Acetate	31.2^a^	19.2^b^	33.9^a^	1.657	0.001
Propionate	12.7^a^	5.84^b^	11.3^ab^	1.487	0.047
Isobutyric acid	0.70	0.81	1.02	0.130	0.370
Butyrate	1.69^b^	2.21^b^	3.98^a^	0.224	0.001
Isovaleric acid	1.25	1.41	1.84	0.338	0.543
Valeric acid	0.31	0.40	0.58	0.079	0.219
Ammonia-nitrogen, mg/L	24.4^ab^	26.1^a^	19.4^b^	1.673	0.048
Microbial protein, μg/ml	457^a^	420^b^	450^a^	7.85	0.027

^1^GN, growth normal lambs; GR, growth-retarded lambs; GRP, growth-retarded lambs supplemented with probiotic. ^a,b^ Mean values in columns without a common letter are significantly different (P < 0.05).

### 3.4. Ruminal microbiota diversity of growth-retarded lambs

**Figure 2:**
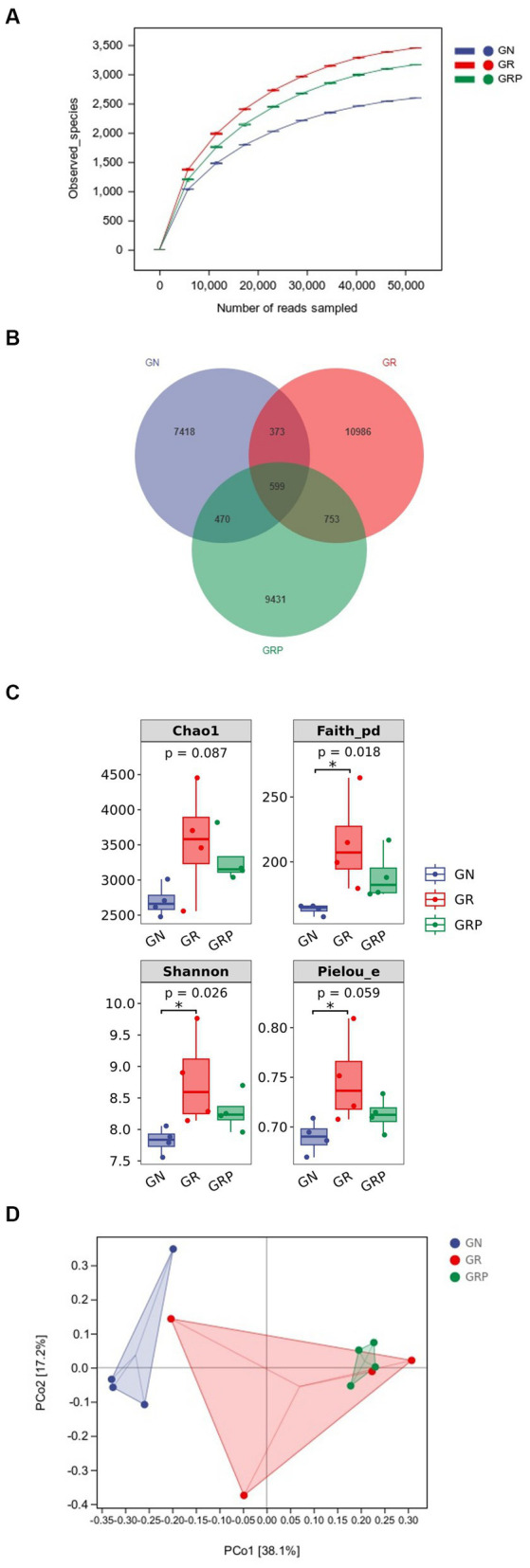
Effect of probiotic supplementation on the ruminal microbial alpha diversity of growth-retarded lambs. **(A)** Rarefaction; **(B)** Venn diagram based on the genus-level diversity; **(C)** the ruminal microbiota diversity estimated by Chao 1, Faith_pd, Shannon and Pielou_e; **(D)** Principal coordinate analysis (PCoA) based on Weighted UniFrac distance. *p < 0.05. GN, growth normal lambs; GR, growth-retarded lambs; GRP, growth-retarded lambs supplemented with probiotic.

In total, 1,682,519 raw reads were generated from 12 rumen fluid samples of lambs, and 746,595 effective reads were obtained after quality control, with an average of 62,216 ± 3,701 reads per sample. As showed in Figure 2A, the rarefaction curves revealed that there were sufficient microbial sequences to accurately describe the ruminal bacterial composition of each group. Based on 97% similarity, 36,880 ASVs were annotated, among which there were 599 shared bacterial ASVs (Figure 2B).

Compared to the GN group, lambs from the GR group exhibited a higher (*p* < 0.05) alpha diversity in the rumen, as evidenced by the increased Shannon, Faith_pd and Pielou_evalues (Figure 2C). In the rumen, principal component analysis revealed that the PCo1 and PCo2 explained 38.1 and 17.2% of the variation among samples, respectively (Figure 2D). As showed in the PCoA figure, the plots for different groups displayed obvious divergence. Difference in bacterial community composition were found between the GN and GR groups (*R* = 0.396, *p* = 0.049) and the GN and GRP groups (*R* = 0.968, *p* = 0.032). However, there was no difference between the GR and GRP groups (*R* = −0.042, *p* = 0.548).

### 3.5. Ruminal microbiota composition of growth-retarded lambs

**Figure 3:**
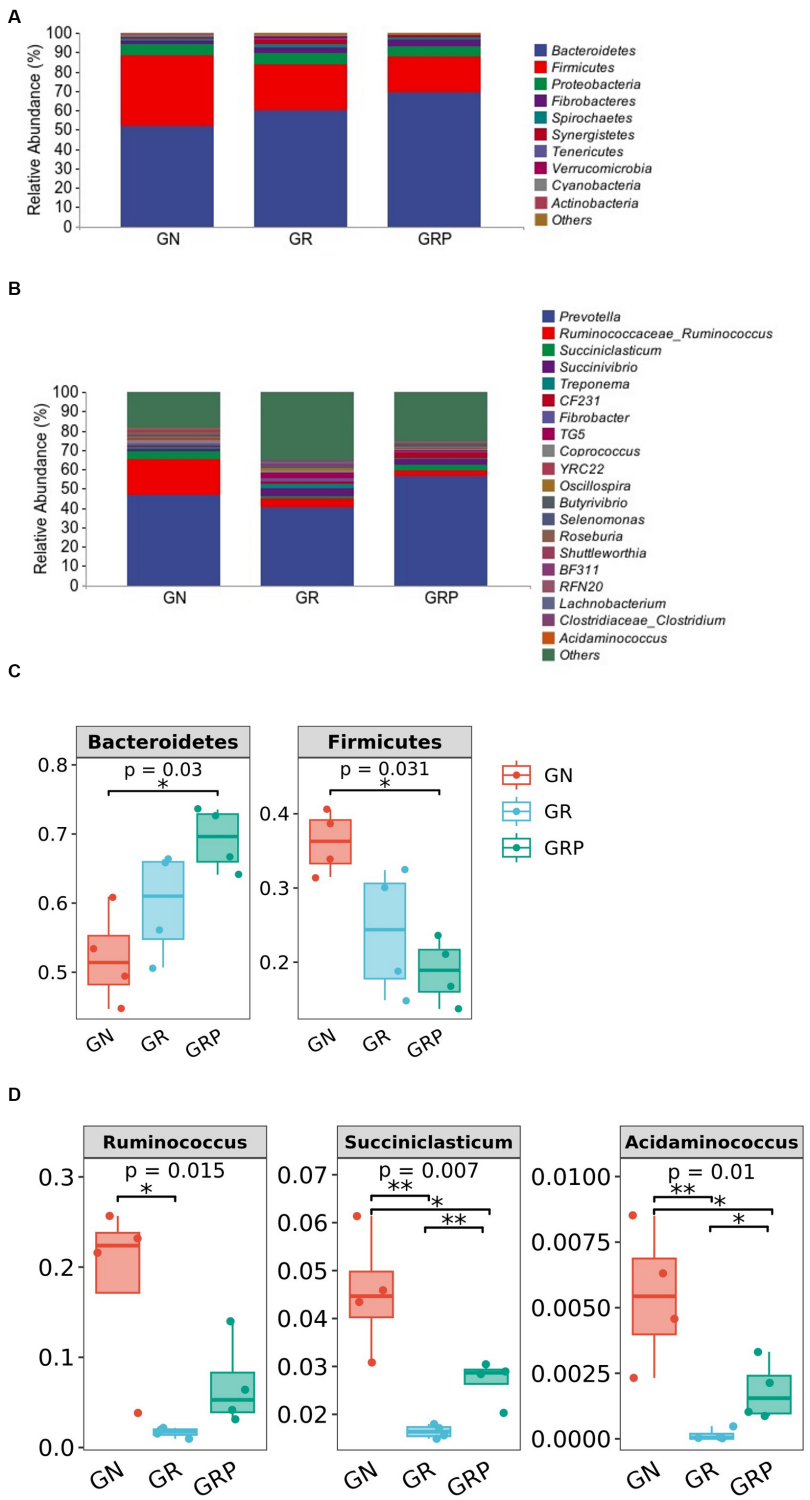
Ruminal microbiota community composition of growth-retarded sheep. Abundances of the ruminal microbiota at the phylum **(A)** and genus **(B)** levels of growth-retarded lambs. The top 10 phyla and the top 20 genera are listed. Comparisons of relative abundances at the phylum **(C)** and genus **(D)** levels were analyzed by the Kruskal–Wallis rank-sum test. *p < 0.05, **p < 0.01. GN, growth normal lambs; GR, growth-retarded lambs; GRP, growth-retarded lambs supplemented with probiotic.

At the phylum level, *Bacteroidetes*, *Firmicutes*, *Proteobacteria*, and *Fibrobacteres* were the four predominant phyla, representing 60.4, 26.3, 5.6, and 2.8% of the total ASVs, respectively (Figure 3A). The relative abundance of *Bacteroidetes* was higher (*p* < 0.05; Figure 3C), but that of *Firmicutes* was lower (*p* < 0.05; Figure 3C) in the GRP group than in the GN group.

As showed in Figure 3B, there were 20 most dominant microbial genera in the rumen of lambs. The relative abundances of *Ruminococcus*, *Succiniclasticum,* and *Acidaminococcus* were the lowest (*p* < 0.05; Figure 3D) in the GR group. However, supplementation with probiotic increased the relative abundances of these three genera.

**Figure 4:**
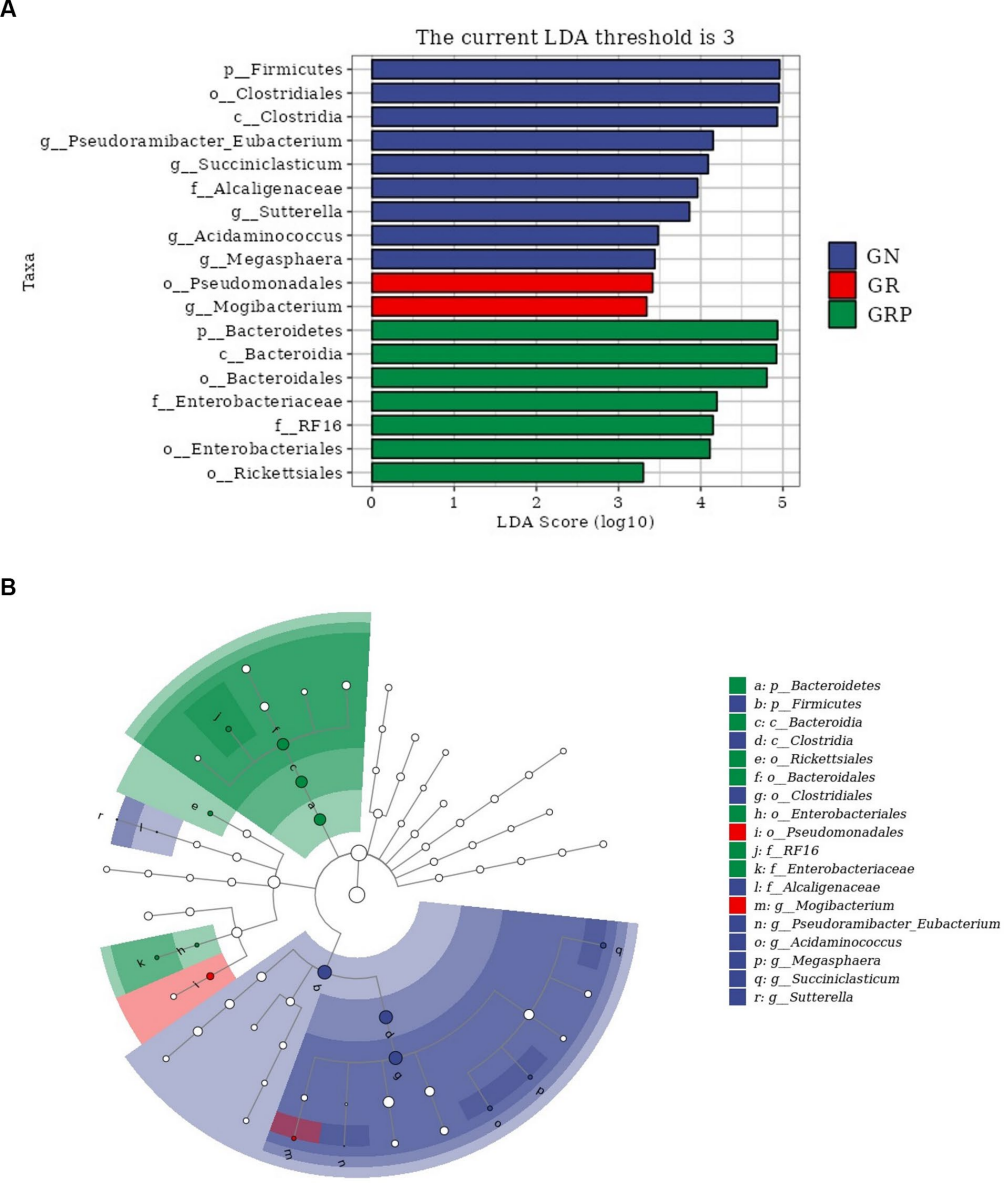
The ruminal microbial community composition modulated by probiotic supplementation and key phylotypes of the ruminal microbiota among the three groups (*n* = 4). **(A)** Linear discriminant analysis (LDA) score. Taxa with LDA score greater than 3 are shown in the histogram. The greater the LDA score was, the more significant the phylotype microbiota was in the comparison. **(B)** Linear discriminate analysis effect size taxonomic cladogram. The colored nodes from the inner circle to the outer circle represent the hierarchical relationships of all taxa from the phylum to the genus level. Taxa enriched in the GN group are shown in blue, taxa enriched in the GR group are shown in red, taxa enriched in the GRP group are shown in green, and taxa with nonsignificant changes are shown in white. The diameter of each small circle represents the taxa abundance.

To identify the key phylotypes of ruminal microbiota among the three groups, LEfSe analysis was performed (Figure 4A). The LDA score results showed nine discriminative features in the GN group, and *Firmicutes*, *Clostridiales* and *Clostridia* were the main microbiota. The GR group showed two dominant microorganisms, which were *Megasphaera* and *Mogibacterium.* The GRP group showed seven discriminative features, and the major microbiota were *Bacteroidia*, *Bacteroideres,* and *Bacteroidales*. An evolutionary clustering analysis diagram was constructed to identify major microflora by taxonomy (Figure 4B). In the cladogram, *Bacteroides* was the richest in the green areas, and *Firmicutes* had the highest abundance in the blue area, which represented the GRP and GN groups, respectively. These results inferred that probiotic supplementation altered the key phylotypes of the ruminal microbiota in growth-retarded lambs and promoted the multiplication of specific bacteria.

### 3.6. Relationship between ruminal microbiota and phenotypic variables

**Figure 5:**
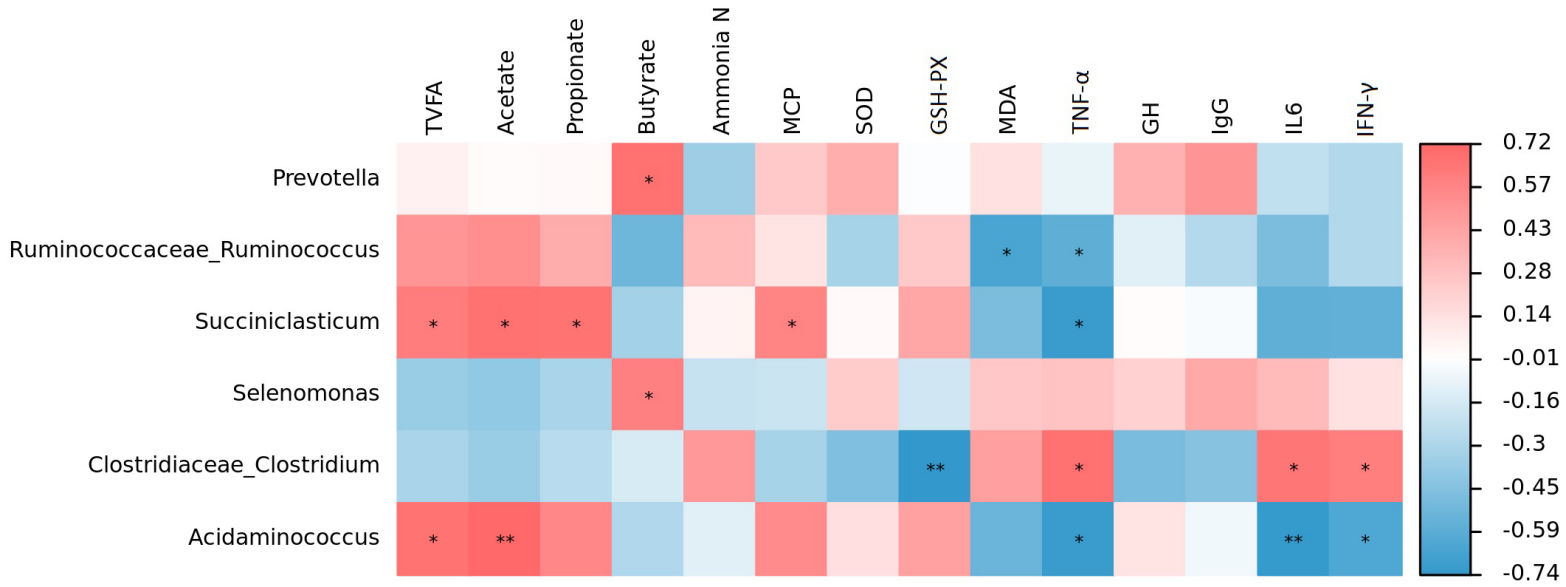
Correlation analysis between ruminal bacteria and phenotypic variables. Red represents a positive correlation and blue represents a negative correlation, the intensity of the color indicates the degree of correlation coefficient. *p < 0.05 and **p < 0.01.

Spearman correlation analysis revealed that *Prevotella* was positively correlated with butyrate (*p* < 0.05; Figure 5). Similar relationships were found between *Succiniclasticum* and total VFA (*p* < 0.05), acetate (*p* < 0.05), propionate (*p* < 0.05) and MCP (*p* < 0.05), but an opposite relationship was observed between *Succiniclasticum* and TNF-α (*p* < 0.05). Furthermore, *Acidaminococcus* was positively correlated with total VFA (*p* < 0.05) and acetate (*p* < 0.01), but negatively correlated with TNF-α (*p* < 0.05), IL-6 (*p* < 0.01) and IFN-γ (*p* < 0.05). *Selenomonas* displayed a positive correlation with butyrate (*p* < 0.05). *Clostridiaceae_Clostridium* was negatively correlated with GSH-Px (*p* < 0.01) but positively correlated with TNF-α (*p* < 0.05), IL-6 (*p* < 0.05) and IFN-γ (*p* < 0.05). Additionally, *Ruminococcaceae_Ruminococcus* was negatively correlated with MDA (*p* < 0.05) and TNF-α (*p* < 0.05).

## 4. Discussion

Lambs with growth retardation have higher morbidity and mortality and lower growth performance. No surprise, 4 growth-retarded lambs died during the feeding trial and the growth rate of growth-retarded lambs was slower than that of normal lambs in the current study. Ma et al. (2021b) reported the same findings in growth-retarded yaks. The positive effects on ADG and DMI indicated that dietary supplementation with probiotic was beneficial to improve growth-retarded lamb growth. Abdel-Wahed et al. (2022) found that both *Bacillus* sp. and *Lactobacillus* sp. can be used as probiotics to achieve therapeutic purposes, such as increased the growth performance of ewes and reduced the incidence of diarrhea in their newborn lambs (Khattab et al., 2020). Also, the results of a previous experiment using the same probiotic showed that it could enhance the growth performance of piglets (Xie et al., 2022). For ruminants, Ossimi lambs that received *Lactobacillus* sp. showed the highest weight gain (El-Ashker et al., 2018). Khattab et al. (2020) reported that probiotic bacteria (*B. subtilis* and *L. casei*) supplementation enhanced the weaning weight, average body weight gain and the health status of the lambs. Another report showed that *Lactobacillus acidophilus* supplementation enhanced the feed intake and daily weight gain of Nubian goats (Azzaz et al., 2015). The positive effect of probiotics supplementation may contribute to the stabilization of gastrointestinal microbiota, which promotes growth and enhances the host resistance to diseases (Xue et al., 2011; Du et al., 2018).

Various stressful conditions, such as malnutrition during the neonatal periods and illness followed by coughing, broken the balance between antioxidant defense system and free radical generation system. The lower concentrations of SOD and GSH-Px and the higher level of MDA in the plasma of growth-retarded lambs than normal lambs indicated that the animals were in a state of oxidative stress, therefore, the growth-retarded lambs had lower growth performance. With probiotic supplementation, the oxidative stress of growth-retarded lambs was alleviated, which was evidenced by the increased SOD and GSH-Px activities and the decreased MDA content. It most likely that probiotics can scavenge excess free radicals and improve the antioxidant capacity of animals (Amaretti et al., 2013). Using *Lactobacillus farciminis* was also reported to affect the antioxidant status of lambs positively (Aydin et al., 2021).

Immunoglobulin is a nonspecific immune molecule in animals (Chen et al., 2021), and IgA, IgM, and IgG are the three main types of immunoglobulins in blood. In the current study, lower concentrations of plasma IgG in growth-retarded lambs suggested that their immune function was less developed than normal lambs. Low immune function may have made growth-retarded lambs more susceptible to disease, which could lead to higher morbidity and mortality (4 growth-retarded lambs died during the feeding trial). Previous studies reported that growth-retardation animals have higher levels of the proinflammatory cytokines TNF-α and IL-6 in blood than growth-normal animals (Zhang et al., 2018; Ma et al., 2021a). In line with these studies, growth-retarded lambs exhibited a higher inflammatory response compared to normal lambs in this study. However, probiotic supplementation ameliorated the inflammatory response of growth-retarded lambs, which was evidenced by increased IgG and decreased levels of the pro-inflammatory cytokines IL-6 and TNF-α. Chen et al. (2022) found that dietary supplementation with the same probiotic product as our study (*L. acidophilus* and *B. subtilis*) could increase IgG and decrease IL-6, TNF-α and IFN-γ in the intestines of laying hens. Some studies reported that probiotics treatment enhanced the concentration of IgG in the serum of lambs (Jia et al., 2018; Chen et al., 2021). *Lactobacillus delbruekii* supplements could be a useful nutritional support for the immune system in healthy lambs (El-Ashker et al., 2018). Altmeyer et al. (2014) inferred that any factors could modify the gut situation will influence immunity, because there were a large number of immune cells in the gastrointestinal tract.

Volatile fatty acid, an energy source of ruminant, contribute to ruminant health by sustaining the rumen ecosystem. It is well documented that rumen VFA, especially butyrate, are the primary chemical stimulators of rumen development (Plöger et al., 2012; Wang et al., 2016). The present results concerning reducing the total VFA with the GR group indicated that growth-retarded lambs had poorer rumen fermentation than normal lambs. In line with this study, growth-retarded yaks have lower rumen VFA fermentation than normal yaks (Hu et al., 2019). Furthermore, the increased concentrations of total VFA and butyrate in the rumen of growth-retarded lambs through the supplementation with probiotic might have beneficial effects on the rumen development of these lambs, which could then recover the growth rate. A meta-analysis by Frizzo et al. (2011) reported that probiotic supplementation could increase the feed digestion, promote the synthesis of VFA, and enhance the growth performance of cattle. Probiotics were also used to maintain gut health and improve feed efficiency by increasing the absorption rate of volatile fatty acids (Krehbiel et al., 2003). In addition, the rumen NH_3_-N concentration decreased in the GRP group, which indicated that the diet supplemented with probiotic product improved NH_3_-N utilization. Similar results revealed that the supplementation with probiotic decreased the NH_3_-N concentration of lambs (Hassan et al., 2020; Chen et al., 2021). Ammonia-N in the rumen is the main nitrogen source for the synthesis of microbial protein, and the declining of NH_3_-N in the GRP group is in line with the increase in MCP concentration. Probiotics product can improve stimulation of ruminal microorganism activity by assimilation of NH_3_-N into microbial protein (Gado et al., 2011).

In this study, the rumen bacterial communities of normal lambs and growth-retarded lambs were compared, and the results of PCoA indicated that the composition and structure of rumen bacterial were distinct between the GN and GR groups. In line with our findings, some studies have reported that the gastrointestinal composition differs between growth-retarded and normal animals (Hu et al., 2019; Ma et al., 2020b), suggesting that the gastrointestinal bacteria can affect the growth of animals. It was also found that the probiotic supplementation group seemed to be comingled together and tended to separate from the GR group. As compared to the factor of host species, dietary factors made more markedly effect on the rumen microflora (Henderson et al., 2015). Du et al. (2018) fed a *B. amyloliquefaciens/B. subtilis* diet to growth-retarded calves aged 3–6 months for 30 days, resulted in more stable of bacterial structure. In the current study, probiotics were used to intervene in the growth of growth-retarded lambs. Chaucheyras-Durand and Durand (2010) found that antibacterial compounds produced by probiotics, such as organic acids, hydrogen peroxide, bacteriocins, and biosurfactants, can inhibit the growth of pathogenic microorganisms. *Lactobacillus*, which is the main component of probiotic products, is an essential member of the intestinal microbiota. It has positive effect on the ruminal environment by promoting the growth of lactic acid utilizing bacteria, and then provide an optimum condition for ruminal microorganisms (Cherdthong et al., 2021). Moreover, as another main component, *Bacillus subtilis* is believed to affect the composition and function of microbial communities through the production of antimicrobials, promoting the growth of beneficial microbes and overall gut health (Luise et al., 2019; He et al., 2020).

*Bacteroidetes* and *Firmicutes* were the dominant bacteria in the rumen of lambs in this study. Ley et al. (2006) found that *Firmicute*s performed an important function on the energy absorption. Furthermore, *Firmicutes* are also reported to play a key role in the degradation of oligosaccharides, starch, and cellulose (Ahmad et al., 2020). Ma et al. (2020b) found that growth-retarded yaks with low feed efficiency exhibited lower relative abundance of *Firmicutes* in the rumen. In this study, growth-retarded lambs had a lower *Firmicutes* relative abundance, which was correlated with their lower growth performance. At the genus level, *Prevotella*, *Ruminococcus* and *Succiniclasticum* were the dominant bacteria in the three groups. The members of *Ruminococcus*, which can produce all organic acids, are cellulolytic, fiber-degrading bacteria (Ezaki, 2015). Li and Guan (2017) found that beef cattle with higher feed efficiency exhibited higher relative abundance of *Ruminococcus* in the rumen. Compared with the GN group, we observed lower *Ruminococcus* relative abundance in the GR group, indicating that growth-retarded lambs had a lower ability to degrade fiber. However, *Ruminococcus* tended to be increased by probiotic supplementation, this result suggested the positive effects of probiotic supplementation on microbial changes and might improve rumen digestion. Van Gylswyk (1995) revealed that *Succiniclasticum* play an important role on the succinic acid production and can convert succinic acid to propionic acid, a major ruminal short chain fatty acid. The relative abundance of *Succiniclasticum* was the lowest in the GR group, but supplementation with probiotic increased its abundance. This change was consistent with the findings of VFA concentration. Therefore, it was inferred that dietary supplementation with probiotics could improve rumen fermentation, which was in line with the increases in total VFA concentration and growth performance of growth-retarded lambs.

The VFAs, especially butyrate, could stimulate rumen epithelial metabolism (Baldwin and McLeod, 2000; Górka et al., 2011). The butyrate concentration was closely positively correlated with the relative abundances of *Prevotella* and *Selenomonas.* It was also found that *Succiniclasticum* and *Acidaminococcus* were positively correlated with total VFA and acetate concentrations, which could explain our previous findings of higher total VFA and acetate in the GRP group. Hence, the ruminal epithelium functional deficiencies of growth-retarded lambs could be repaired by nutritional interventions, which was attribute to the increase of beneficial bacteria abundance and the VFA concentrations (especially butyrate production) in the rumen.

## 5. Conclusion

In summary, the results obtained in the present study provide evidence that dietary supplementation with probiotic (co-fermented by *Lactobacillus acidophilus* and *Bacillus subtilis*) contributes to a number of ways to improve growth performance of growth-retarded lambs, including improvements in antioxidant ability, a decrease in the inflammatory response, and better regulation of the rumen microbiota. These findings may help sheep farmers use nutritional interventions to promote the compensatory growth of growth-retarded lambs.

## Data availability statement

The ruminal bacterial 16S rRNA gene sequencing data have been deposited in NCBI Sequence Read Archive database (accession number: PRJNA857331).

## Ethics statement

The animal study was reviewed and approved by All animal research procedures were approved by the Animal Use and Care Committee, Zhejiang A&F University (ZAFUAC2016002, Hangzhou, China).

## Author contributions

HM: conceptualization, methodology, writing–original draft preparation, writing–review, editing, and funding acquisition. WJ: investigation, data collection, sample analysis, and writing–original draft preparation. YY: investigation, data collection, and sample analysis. YZ: investigation and data collection. ZL: conceptualization. CW: conceptualization, methodology, formal statistical analysis, and funding acquisition. All authors read and agreed to the published version of the manuscript.

## Funding

This research was supported by the fund for the National Natural Science Foundation of China (31902179 and 32172742) and Zhejiang Provincial Cooperative Promotion Plan of Major Agricultural Technologies (2021XTTGXM05-4).

## Conflict of interest

ZL was employed by the company Hangzhou King Techina Feed Co., Ltd.

The remaining authors declare that the research was conducted in the absence of any commercial or financial relationships that could be construed as a potential conflict of interest.

## Publisher’s note

All claims expressed in this article are solely those of the authors and do not necessarily represent those of their affiliated organizations, or those of the publisher, the editors and the reviewers. Any product that may be evaluated in this article, or claim that may be made by its manufacturer, is not guaranteed or endorsed by the publisher.

## Footnotes

^1^https://www.genescloud.cn

## Abbreviations

ADF: acid detergent fiber
ADG: average daily gain
BW: body weight
CP: crude protein
DM: dry matter
DMI: dry matter intake
GH: growth hormone
GR: growth-retarded
GSH-Px: glutathione peroxidase
IgG: immunoglobulin G
INF-γ: interferon gamma
IL-6: interleukin-6
MCP: microbial protein
MDA: malondialdehyde
NDF: neutral detergent fiber
NH_3_-N: ammonia-N
SOD: superoxide dismutase
T-AOC: total antioxidant capacity
TNF-α: tumor necrosis factor alpha
VFA: volatile fatty acid
